# Acceptance, experiences, and needs of hospitalized pregnant women toward an electronic mindfulness-based intervention: A pilot mixed-methods study

**DOI:** 10.3389/fpsyt.2022.939577

**Published:** 2022-08-22

**Authors:** Claudia Schiele, Maren Goetz, Kathrin Hassdenteufel, Mitho Müller, Johanna Graf, Stephan Zipfel, Stephanie Wallwiener

**Affiliations:** ^1^Department of Obstetrics and Gynecology, University of Heidelberg, Heidelberg, Germany; ^2^Department of General Pediatrics, University of Heidelberg, Heidelberg, Germany; ^3^Department of Psychology, Ludwig Maximilian University, Munich, Germany; ^4^Department of Psychosomatic Medicine and Psychotherapy, University Hospital Tübingen, Tübingen, Germany

**Keywords:** digital intervention, mindfulness-based intervention (MBI), obstetric risk, depression, anxiety, mental health, pregnancy, hospitalization

## Abstract

**Background:**

Anxiety disorders and depression during pregnancy are highly prevalent. Hospitalized pregnant women with high maternal or fetal risk represent a particularly vulnerable population often excluded from research samples. Screening for mental health disorders is not routinely offered for this particular patient group. Electronic mindfulness-based interventions constitute an accessible, convenient, and cost-effective mental health resource but have not yet been evaluated for acceptance in inpatient settings. To date, little is known about the needs and perceptions of this group of women.

**Objective:**

The aim of this study was to examine whether a brief electronic mindfulness-based intervention (eMBI) is accepted among hospitalized high-risk pregnant women. We assessed personal motivation and barriers, experiences, usability requirements, and overall acceptance of the eMBI, as well the specific needs and demands of patients with high-risk pregnancies regarding mental health services.

**Methods:**

An exploratory pilot study with a mixed-methods study design was carried out among 30 women hospitalized with a high obstetric risk. The study participants were given access to an eMBI with a 1-week mindfulness program on how to deal with stress, anxiety, and depressive symptoms. Semi-structured interviews were conducted with the 30 participants and analyzed using systematic content analysis. In addition, acceptance and usability were assessed *via* questionnaires.

**Results:**

Study findings showed a high level of acceptance of the eMBI. Most of the respondents were satisfied with the usability and considered the eMBI program to be helpful. The greatest barriers to using the eMBI were a general negative attitude toward using apps, preference for personal contact, or no current need for psychotherapy. Participants criticized the lack of awareness of mental health issues during pregnancy and expressed a need for low-threshold treatment offers, especially during hospitalization.

**Conclusions:**

There is a strong need for mental health services in pregnancy care, especially for pregnant women with risk profiles. An eMBI offers an acceptable means of providing mental health support for hospitalized women with a severe obstetric risk.

## Introduction

Mental health problems are a common phenomenon in the perinatal period. A recent large-scale observational database study found a prevalence of 9.3% for prenatal depression and 16.9% for prenatal anxiety disorders in Germany (1). In addition, the COVID-19 pandemic has had a significant impact on the mental health of pregnant women, with more than one-third reporting clinically relevant symptoms of depression and more than half of women reporting clinically relevant symptoms of anxiety (2–4).

Few studies have examined the prevalence of mental disorders in pregnant women hospitalized for severe maternal or fetal risk. Existing studies suggest that these women represent a particularly vulnerable group with prevalence rates of up to 39–44% for symptoms of anxiety and depression (5–8). One study found that women experiencing a high-risk pregnancy had a five to seven times higher risk for the prenatal onset of anxiety disorders than low-risk patient samples (9).

Women who are hospitalized for pregnancy complications experience stress from being hospitalized in addition to the stress of their high-risk pregnancy (10). Health concerns, loss of control, uncertainty, isolation, separation from home, bed rest, physical discomfort, and obstetric treatments that are often physically and psychologically invasive constitute important stressors (11, 12). Moreover, symptoms of depression and anxiety have been found to be exacerbated by higher levels of stress (13–15).

Depression and anxiety during pregnancy are associated with negative outcomes for both mother and child (16–18). For the fetus, depression is associated with adverse effects through the increased risk of preterm birth, low birth weight, and for being small for gestational age (19–21). Furthermore, prenatal depression appears to affect infant and child development and may increase the risk of developing depression in adulthood (17, 22). Considering the high prevalence and possible adverse outcomes, early detection and effective treatment of these conditions in the peripartum period are essential.

The treatment of peripartum depression and anxiety comprises both drug treatments and psychotherapeutic approaches. Although pharmacological treatment is effective and can be safely utilized during pregnancy under medical supervision, only 2–8% of pregnant women are currently estimated to use antidepressants (23–26).

Pregnant women are particularly reluctant to take medication because of potential risks to the developing fetus; hence, only one-third of pregnant women would consider pharmacotherapy an acceptable treatment option (27). Psychotherapeutic interventions such as interpersonal psychotherapy and cognitive behavioral therapy (CBT), which have been shown to be effective in treating perinatal depression and anxiety (28, 29), are preferred (27, 30, 31). Currently, CBT is the treatment of choice for mental illnesses (28, 32). Yet, reported referral rates for psychiatric consultations in inpatient obstetric samples amounted to only 0.1–0.3% (33, 34).

A number of factors prevent patients from seeking help. In the absence of systematic screening in routine prenatal care, affected women are often not identified (35–38). Many of the signs of perinatal depression, such as fatigue, persistent anxiety, and difficulty in concentrating or sleeping, may overlap with typical “pregnancy symptoms”. Stigma is also considered a major barrier to disclosure and seeking help (27, 39, 40). Other perceived barriers to treatment are difficulties in arranging childcare, transportation, and lack of time (27, 30, 39). Some of these barriers may be overcome through app-based interventions. In fact, app-based interventions could provide low-threshold and stigma-free access at any time and place and maintain anonymity.

Mindfulness-based interventions (MBIs) have recently come into focus as effective treatments for reducing symptoms of depression, anxiety, and stress in the general population (41, 42) and in pregnant women (43–47), combining elements of CBT and psychoeducational content. According to a recent meta-analysis, there was no significant difference between MBI and CBT in treatment success for anxiety and depression (42). Given the increasing omnipresence of the Internet, MBIs can also be offered electronically. Indeed, the field of electronic MBIs (eMBIs) is booming, and a recent meta-analysis showed that eMBIs have a positive impact on mental health outcomes (48). Moreover, eMBIs can be beneficial for a wide range of populations and symptoms (48, 49). Regarding the treatment of depression and anxiety, the effect of eMBIs appears to be similar to MBIs in the traditional face-to-face format (48).

Although women have expressed interest and a desire for electronic interventions in the perinatal period (50), evidence supporting the effectiveness of eMBIs during this time is still lacking (51). Few existing studies show that eMBIs may be beneficial (51), feasible, and accepted (52–54) in the treatment of depression and anxiety in the perinatal period. There is evidence that women with elevated levels of depression, anxiety, or stress during this time may benefit more from MBIs than universal perinatal populations (55), suggesting a promising use in hospitalized women with obstetric risk. Indeed, eMBIs may be particularly useful in the perinatal period because this population faces accessibility issues; in particular, mobility is often limited in women with pregnancy complications.

Women hospitalized due to severe obstetric complications are at risk for depression and anxiety with negative implications for mother and child. To date, little is known about their attitudes and preferences or any barriers to an eMBI. The aim of this qualitative study was therefore to analyze (1) acceptance, personal motivation, and barriers to using an eMBI, (2) the specific needs and demands of patients with high-risk pregnancies regarding mental health services, and (3) usability requirements of the eMBI.

## Methods

### Sample and study design

The study was conducted at the Heidelberg University Women's Hospital, a perinatal center of the highest level. Study participants were recruited between January and May 2019. Eligibility criteria for participation included a gestational age between ≥24 and ≤ 34 weeks, age of 18 years or older, German language fluency, and admission to the obstetrics ward. Women were not eligible to participate if they were expecting multiples. The subsample for qualitative analysis included 30 pregnant inpatient women. This sample was part of a larger pilot study within the mindmom study (56) with a mixed-method design, aiming to investigate the effectiveness of a 1-week mindfulness course on prenatal depression and anxiety in hospitalized obstetric patients (57). A total of 107 randomly selected inpatients were approached: 22 declined (22/107, 21%), 85 agreed (85/107, 79%), and 68 were finally enrolled (68/107, 64%). Reasons for not participating included lack of interest or time and severe pregnancy complications.

### The electronic mindfulness-based intervention

The participants took part in an eMBI using a shortened version of the mindmom program, which is currently being investigated in a prospective randomized trial (56). The app was developed by an interdisciplinary team of gynecologists and psychologists and consists of 8 weekly modules. These modules are designed to help manage stress, pregnancy-related anxiety, and symptoms of depression by providing pregnancy-related mindfulness exercises, psychoeducational content and CBT approaches. With integrated mindfulness and CBT exercises, the pregnant women train their own perception of needs in order to apply these skills specifically to their own needs in the sense of a mindful interaction with themselves and the unborn child. The app contains instructional videos, audio files, and interactive worksheets.

In this study, the time frame was tightened to a 1-week program on mindfulness. After obtaining informed consent, participants were provided with a tablet and wireless internet service and were given access to the app. The program consisted of three 45-min modules which were provided on Day 1 (“fears and worries about birth and parenting”), Day 3 (“coping with stress”), and Day 5 (“me and my baby”) of the study for participants to complete. Table 1 provides an overview of the structure and content of the modules. Completed modules were accessible at any time.

**Table 1:** Structure and content of the modules.

Topics	“Fears and worries about birth and parenting”, “coping with stress” and “me and my baby”
Psychoeducational content	Knowledge transfer, for example about stress and its effects on the body, about the emergence of mental vicious circles or the importance of individual sources of strength
Practical skills	Practical exercises and guidance on how to manage stress and anxiety, exit vicious circles through individual sources of strength, encouraging sentences and reward cards
Mindfulness exercise	Completing a mindfulness exercise such as mindful breathing, a mindful body scan or mindful “loving kindness”

### Measurements

All questionnaires were filled out electronically. Participants were asked to complete a baseline questionnaire on sociodemographic data, medical and psychiatric history, and internet usage behavior before starting the 1-week eMBI program. These data were cross-checked with hospital records. After completing the study, participants were asked to answer a final questionnaire about their experience with the mindmom app and their demands for mental health care services during pregnancy.

We used qualitative methods to gain a deeper understanding of pregnant women's perspectives and needs through first-hand experiences and truthful quotes from semi-structured interviews. The interviews were conducted after completing the first module and focused on experiences, needs and acceptance of pregnant women, as well as the usability of the app. The interviews were conducted by a trained interviewer under supervision of a senior physician following an established interview guide with 20 open-ended questions. The interviews were recorded with permission of the interviewees and lasted 10 min and 32 s on average (range 5–19 min). The main themes discussed were usability requirements, acceptance and motivation to use an eMBI, and patients' needs and experiences.

### Data analysis

After the interview process was completed, the audio records were transcribed verbatim and verified against the actual recordings and notes taken during the interviews. Transcripts were uploaded into the MAXQDA qualitative data analysis software (MAXQDA Plus 2020, Release 20.4.0) and analyzed using systematic qualitative content analysis (58, 59).

This analysis process was carried out in three main phases: preparation, organizing, and reporting. In the preparation phase, the authors began to familiarize themselves with the data by going through the transcripts several times. In the organization phase, a coding scheme was created based on the interview questions to organize the data into categories, and the transcripts were coded using this coding scheme. To refine the understanding of the material, unexpected content was also considered, and the coding scheme was supplemented with categories extracted from the material. This is referred to as a mixed deductive-inductive approach (58). To enhance the trustworthiness and credibility of the data analysis, representative quotes are provided (60), which have been translated into English. In addition, the results from the qualitative analysis were compared with the quantitative measures from the questionnaire; this validation strategy is also referred to as triangulation (61). The final phase involved reporting on the analysis process and results (58).

## Results

### Demographics

In all, 30 pregnant women were included in the study sample. Semi-structured interviews were conducted with 30 women and 21 women (21/30, 70%) completed the final questionnaire. Mean gestational age was 30 weeks among the women interviewed (SD 3.6; range 25–34). The characteristics of this sample are summarized in Table 2, and the most common diagnoses are shown in Table 3. In all, 29 women (29/30, 97%) used the Internet often or very often and 29 women (29/30, 97%) used the smartphone often or very often. Regarding information gathering, 29 women (29/30, 97%) frequently used the Internet to inform themselves about pregnancy-related topics, while 22 women (22/30, 73%) also used a pregnancy mobile app.

**Table 2:** Sample characteristics (***n*** = 30).

**Characteristic**	**Value**
Age in years, mean (SD)	31.7 (5)
**Civil status**, ***n*** **(%)**
Married and living together	20 (67)
Married and living apart	0 (0)
Single	10 (33)
**Education level**, ***n*** **(%)**
Lower secondary qualification	2 (7)
Higher secondary qualification	7 (23)
University entrance qualification	21 (70)
**Number of children**, ***n*** **(%)**
0	21 (70)
1	8 (27)
≥2	1 (3)
**Income**, ***n*** **(%)**
< € 1,500 (US $1,633.44)	6 (21)^a^
€1,500–€4,999 (US $1,633.44–US $5,443.71)	19 (66)^a^
>€5,000 (US $5,444.8)	4 (14)^a^
**Gravidity**, ***n*** **(%)**
1	15 (50)
2	12 (40)
≥3	3 (10)
**Mental illness**, ***n*** **(%)**
Current mental illness	0 (0)^a^
Previous prepartum depression and/or anxiety	4 (13)
Previous postpartum depression and/or anxiety	1 (3)
**Mental illness treatment**, ***n*** **(%)**
Previous outpatient psychotherapeutic treatment	6 (20)
Previous inpatient psychotherapeutic treatment	2 (7)
Medication	0 (0)
Previous medication	7 (23)

a *n = 29*.

**Table 3:** Diagnoses at admission (*n* = 30, multiple answers possible).

**Diagnoses**	***n* (%)**
Prelabor rupture of membranes/premature labor	13 (43)
Cervical insufficiency	7 (23)
Intrauterine growth restriction /oligohydramnios	5 (16)
Vaginal bleeding	5 (16)
Fetal malformation	3 (10)
Infections	3 (10)
Polyhydramnios	2 (7)
Placental disorder	2 (7)
Gestational diabetes	1 (3)
Other	4 (13)

### Interview findings

After analyzing the data, three main categories and seven subcategories were identified (Table 4):

(1) usability requirements.
(2) acceptance of eMBIs.
(3) demand for mental health support.

**Table 4:** Main categories and subcategories identified through content analysis.

**Main categories**	**Subcategories**
Usability requirements	User feedback
	Suggestions for improvement
Acceptance of electronic mindfulness-based interventions	Motivation with personal barriers and personal facilitators
	Positive experiences
	Negative experiences
Demand for mental health support	Psychological burden during pregnancy
	Demand for support during pregnancy in general and in populations with obstetric risk

#### Usability requirements

##### User feedback

Unanimously, all participants confirmed that the app was user-friendly. The handling was described as intuitive, simple, and self-explanatory. Most of the participants reported no problems navigating or playing the video and audio files and were satisfied with the technical realization. The majority of participants found the content of the texts, audio files, and videos informative, helpful, and entertaining. The simplicity of the design and the discreet color scheme were rated positively. A clear and professional presentation seemed to be particularly important to the patients.

##### Suggestions for improvement

Several participants suggested better adapting the program for cell phones in order to improve handling. Three patients suggested sending an automatic email reminder or push messages to alert participants if new exercises were available. Another suggestion was to set up a forum or live chat to interact with other participants and talk about their concerns.

*It would be great […] if the app had a push function, like a reminder: “Your new module is available” etc. This is certainly not a bad idea. Because otherwise you might simply forget about it. Just like with all the things that make you think “actually, it's really nice to do something like that” and then, when there isn't any reminder or message, you forget about it right away*. [Woman, 31 years; 33rd week of pregnancy]

#### Acceptance of eMBI

##### Motivation: Personal barriers and personal facilitators

The vast majority of patients stated that they would be motivated to continue the mindfulness program for 8 weeks (83%, 25/30). Two patients (7%, 2/30) indicated that they were rather less motivated because they had no current need for additional support. Three patients (10%, 3/30) said they only used apps infrequently or reluctantly. Other reasons for a low motivation were preference for personal contact or lack of time. While many participants found the program helpful and enjoyed using it, some participants commented that not all women would participate in a mindfulness program because they did not want to face their fears and concerns.

*As I said, it was very pleasant and I indeed had the feeling that it helped somehow. […]Even if it is simply because you have the feeling of doing something just for yourself for half an hour. Being able to blend out the stress surrounding you, knowing that you now have half an hour to really focus only and truly on yourself*. [Woman, 24 years, 31st week of pregnancy]

Opinions about using the eMBI in an inpatient setting varied. Seven women reported problems: that they were kept from practicing the exercises by roommates or visitors, that the atmosphere was too noisy and restless, or that they had problems with the wireless connection in the hospital. Others commented that their hospital stay was monotonous and boring, and that the eMBI exercises were a nice change of pace.

Some patients appreciated the low-threshold access of an eMBI. The anonymity and time flexibility were positively emphasized.

*[…] especially for modules that you can enter anytime and that allow a certain flexibility in time, you are perhaps more likely to be interested than for a fixed date, where you would have to pause your everyday life. In this respect, I think it's a big advantage*. [Woman, 34 years, 26th week of pregnancy]

##### Positive experiences

All women described positive experiences with the program. Many patients stated as a positive experience that the exercises had encouraged them to actively think about themselves and their fears. The exercises had made them deal with their fears and worries in a way they might not have done otherwise (70%, 21/30). In addition, it was positively emphasized that the mindfulness program encouraged them to concentrate on themselves, and that they had taken time for this (20%, 6/30). Another positive aspect mentioned was that the exercises helped them to relax and provided strategies for coping with stress (37%, 11/30). Some women stated that the app made them aware that others share the same concerns, hence that they were not alone with their problems and insecurities (23%, 7/30).

Interviewer: Do you think there are women who are not doing so well? Woman: Yes, who are then perhaps more reassured when they realize other people do feel the same way or other people are also insecure or have anxieties. [Woman, 37 years, 32nd week of pregnancy]

The content was described as very appropriate, with women indicating that they recognized themselves there. Furthermore, it was appreciated that the app had a professional medical background compared to other apps available.

##### Negative experiences

Although confronting and dealing with one's own fears and worries was mostly perceived as a positive experience, some patients stated that the confrontation made them feel uncomfortable or imagined that it might make others uncomfortable. One patient stated that an app could not help her manage her worries, as she would prefer face-to-face contact when she was feeling anxious. Finally, three women criticized that the program was not yet fully adapted to inpatient samples, e.g., they were on bed rest due to their pregnancy complications and were therefore unable to practice all the exercises in the program.

*What struck me, well I have to say it's very individual, but the reason I'm here is a cervical insufficiency, which means that I'm not allowed to jump around or do everything the way I usually do it. And then there was this point where it was stated that you can avoid stress by doing things that make you feel good, like sports, going out…that felt like a sting […]*. [Woman, 31 years, 25th week of pregnancy]

#### Demand for mental health support

##### Psychological burden during pregnancy

It was criticized that the media image and the social concept of pregnancy strongly deviated from reality, with the image of the always happy pregnant women still very present.

*[…] so, this common image that is always presented that every pregnancy is so beautiful and that there is nothing more beautiful in the world. And then when you are pregnant yourself and think “well, it isn't as beautiful as they said”*. [Woman, 24 years, 33rd week of pregnancy]

Participants also criticized the lack of awareness of the psychological burden during pregnancy, saying that it was still a taboo subject that needed to be better addressed: Mental health is not given a high priority. As a result, pregnant women often feel misunderstood by their partners or society and, because this issue is not widely thematized, many pregnant women also do not deal with the topic, misjudge themselves, and suppress their own problems and fears. Several participants stated that they believed a large number of cases went unreported. In addition, the pregnant women would often not dare to talk about problems and fears or seek help.

##### Demand for support during pregnancy in general and in populations with obstetric risk

All women agreed that pregnant women who were hospitalized due to pregnancy complications had a special need for support. For these women, concerns about complications and their possible consequences are added to the already existing fears. Several women (47%, 14/30) stated they had been less worried in previous, uncomplicated pregnancies. In the context of pregnancy complications and hospitalization, women reported feelings of shock, uncertainty, loneliness and isolation, frustration with ordered bed rest, and helplessness.

*Yes, definitely. Because I almost have the impression that the demand and need are even higher, as well as the uncertainty. Sure, here they are taking care of you and so on, but I can only say what I'm feeling here, there is definitely a need to talk, because you are simply worried and insecure. Especially for the inpatients, I think it's important*. [Woman, 31 years, 25th week of pregnancy]

It was criticized that concrete offers for mental health support were not included in routine care. Participants stated that just knowing about possible support and low-threshold offerings would be a support in itself.

Women had different requests for mental health services during the peripartum period, including medical or psychological counseling or additional education. While some stated that face-to-face contact was important, most indicated that they could easily imagine support through an app. Many would like to get support through an eMBI: 93% (28/30) of women said there was a great need for an eMBI like the mindmom app among pregnant women.

### Participants' view of the program in the postintervention evaluation

In general, the participants were satisfied with the program. All but one participant would recommend the mindfulness program to other pregnant women. Additionally, 57% (12/21) of the participants would be motivated to continue the program and 57% (12/21) said they had personally benefited from the program. All but 3 participants (18/21, 86%) indicated that, looking back, they wished they had had more support during pregnancy. Participants would have liked additional support to deal with the stress and concerns of pregnancy, provided by midwives (6/21, 29%), gynecological care (3/21, 14%), psychological care (2/21, 10%), support with official matters (e.g., application for parental allowance, financial problems) (13/21, 62%), specific online services for pregnant women (6/21, 29%), and/or the mindmom app (4/21, 19%). Furthermore, the participants stated that the app was easy to navigate (15/21, 71%), the texts were understandable (20/21, 95%), and the exercises were helpful (16/21, 76%).

## Discussion

### Principal results

The primary aim of this study was to examine the acceptance of an eMBI in hospitalized, high-risk pregnant women. Our study showed a high acceptance of eMBIs: the participants stated that they benefited from the program, were motivated to continue, and would recommend the program to others. Several barriers were identified: reluctance to use apps and preference for personal contact, no current need for support, or lack of time. The low inhibition threshold and time flexibility of an electronic program were rated as positive. The program was described as helpful, with several useful strategies identified. Focusing on oneself, learning relaxation techniques, and actively confronting one's own fears and concerns were described as beneficial. One criticism was that the app was not yet fully adapted to women with pregnancy complications because the exercises were not suitable for women with ordered bed rest. Usability was generally rated as positive.

The women stated that psychological stress during pregnancy was an important topic and criticized the lack of awareness of the issue. Most participants expressed a great need for an eMBI like the app used in this study, both for a pregnancy without complications and especially in hospitalized women with severe obstetric risk.

### Comparison with prior work

Our study showed a high acceptance of eMBI among high-risk, hospitalized pregnant women and confirmed the findings of previous studies on the acceptance of mobile or web-based interventions demonstrating high acceptance among women with postpartum depression (PPD) (52, 54). Haga et al. found that nearly 2 of 3 respondents would recommend a web-based intervention for reducing the risk of PPD, and 43% (44/103) indicated that the information presented was relevant to them (54). Our study found that 95% (20/21) of participants would recommend the program to others, 76% found the exercises helpful (16/21), and 57% (12/21) reported having benefited from the eMBI. While the study by Haga et al. focused on pregnant women and mothers and their partners, our study included hospitalized pregnant women with increased fetal or maternal risk. The acceptance rates of the mindfulness program appear to be higher, suggesting that an eMBI has an even better acceptance among this particular population.

In another study, Avalos et al. showed that a mobile mindfulness intervention was well accepted among women with moderate to moderately severe PPD symptoms. In the study, 69% of participants were very or extremely satisfied with the app, and all participants planned to continue their mindfulness practices after the study ended (52). In our study, only 57% (12/21) of participants reported being motivated to continue mindfulness training. This might be explained by the fact that Avalos et al. included only women with elevated depression symptoms in their study. In our study, the women were not screened for depression or anxiety symptoms before enrollment; thus, the study sample comprised women with depression or anxiety symptoms and mentally healthy women alike.

Maloni et al. reported that many women suffering from PPD after pregnancy complications expressed interest in web-based treatment for PPD. Barriers to seeking treatment included lack of time, stigmatization, lack of childcare, not wanting to tell the doctor about PPD, and difficulty in finding help (50). Although that study involved a different population, namely, women with PPD, the need appears to be equally high regardless of gestational age. Women in our study also reported stigmatization and a lack of public awareness and a possibly high number of unreported cases.

The high underreporting of pregnant women with prenatal depression and anxiety disorders is consistent with findings from other studies. Although pregnancy and early motherhood are characterized by regular contact with the health care system, studies show that prenatal mental illness often remains undiagnosed. Buekens et al. found that only one-third of women presenting with depressive symptoms are recognized in gynecologic practice (35). In another study, pregnant women who received prenatal care at a university hospital were screened for psychiatric disorders; however, a corresponding diagnosis was only recorded in the medical charts of 18% of women who screened positive (38). Byatt et al. reported that despite the high prevalence of depression (26.9%) and anxiety symptoms (12.6%), high-risk inpatient pregnant women rarely receive psychological screening and/ or treatment (5%) (62). This supports the need for routine screening for mental disorders in pregnant women in general, and especially in the vulnerable group of high-risk, inpatient pregnant women.

A major barrier to disclosure of perinatal depression symptoms also appears to be the internal and external stigmatization from health care providers and stigma from treatment. Moore et al. found that many women feared feeling like a “bad” or “failed” mother (internal stigma) and feared being stigmatized if they disclosed their symptoms to a health care provider (external stigma). In that study, some participants also described negative experiences after disclosing information to health care providers (40). Gawley et al. also showed that health care students stigmatize people with mental health problems, including pregnant women with depression, pointing to the need for improved education about prenatal depression and its treatment (39).

Other studies found that lack of time was the greatest barrier to seeking treatment for perinatal depression during pregnancy (27). Lack of time was also an important barrier in our study; however, the time flexibility of an eMBI was also positively highlighted. This indicates that an eMBI may represent an important alternative to traditional psychotherapy, especially in a patient group that is as limited in mobility as the group of hospitalized pregnant women. In addition, women who have had a high-risk pregnancy may also experience additional barriers to treatment after delivery because of their own postpartum physical recovery and the medical complications of their children.

In addition to these common barriers, the COVID-19 pandemic has led to an increase in anxiety and depression symptoms during pregnancy, increasing the demand for mental health care. Almeida et al. have shown that pregnant women are particularly at risk for developing mental health problems during the pandemic. Social support appears to be a key protective factor (2). Unfortunately, visitation has been restricted in many hospitals due to the pandemic, which may affect social support for pregnant women at increased maternal and fetal risk who are hospitalized. Hamidia et al. showed that the COVID-19 pandemic may increase the prevalence and persistence of postnatal depression in women with a high-risk pregnancy (4). At the same time, access to in-person psychological treatments has been severely limited in the wake of the COVID-19 pandemic, further highlighting the need for effective interventions delivered remotely (3).

The field of eMBIs is booming. An updated meta-analysis on the impact of eMBIs on mental health from 2021 was able to include 97 RCTs, demonstrating the growing interest in implementing eMBIs. This meta-analysis showed that eMBIs had a significant impact on mental health and confirmed previous evidence that eMBIs were beneficial for a wide range of populations and symptoms (48, 49). However, there are only few studies of pregnancy-specific eMBIs with only small samples and lacking randomized controlled approaches (43, 51, 55, 63). Our study highlights the need for eMBIs to support pregnant women, especially if hospitalized due to severe obstetric risk, and points to the need for larger randomized trials to demonstrate the efficacy and effectiveness of eMBIs in this specific population. Despite the short, 1-week eMBI program, the women in our study reported positive experiences and indicated that they benefited from the program. In fact, a meta-analysis including 65 randomized controlled trials showed that brief mindfulness interventions have an immediate and significant effect on reducing depression, stress, and anxiety in both nonclinical and clinical samples (64).

While the benefits of MBIs have been well established, less attention has been paid to the potential harms. In our study, some patients indicated that they felt uncomfortable when being confronted with their fears and worries or imagined that others might feel uncomfortable. As psychotherapy, pharmacotherapy, physical exercise, and meditation can also cause harm, it is essential to consider negative effects such as increased anxiety and unpleasant experiences through using MBIs (65). However, this issue has not yet been sufficiently explored in the current literature and should be brought more into focus.

### Limitations

Several limitations should be considered. We might not be able to generalize our results to other samples. The average age of the study participants was 31.7 years, which is in line with the data on the average age of women in Germany at birth (31.6 years overall, 30.2 years for the first child) (66). However, the average education of the participants was considerably higher than the average education in Germany. There is evidence that women with a low socioeconomic status are more likely to suffer from depression than women of higher socioeconomic status (67). Goodman et al. found that low-income African American women preferred psychosocial approaches to prevent perinatal depression over pharmacotherapy and rated them as more credible and positive (31). Although their study focused on prevention rather than treatment of depression, the results suggest that women of lower socioeconomic status may also show high levels of acceptance of mindfulness-based interventions. Furthermore, the program was offered only in German and thus non-German speakers were not eligible to participate. Future eMBI studies should cover different languages and a broad socioeconomic spectrum, given the high rates of psychological burden in minority populations (68).

The qualitative interviews were conducted by a member of the study team, which could be considered a confounding variable. However, the results of the interviews were consistent with the results of the quantitative data, which increased the reliability of the results. Moreover, the sample size with *n* = 30 can be considered adequate for a qualitative study setting (69).

Only 21 of the 30 women interviewed completed the final questionnaire (21/30, 70%); however, dropouts are common in most electronic programs (70). Similar studies reported even lower compliance rates from 21 to 38% (51, 71). In our particular study population, the majority of dropouts can be explained by hospital discharge or actually giving birth prematurely before the final assessment.

### Conclusions

This study demonstrates that relieving stress and anxiety through an eMBI seems to be an acceptable option for hospitalized women with a severe obstetric risk, who report increased psychological distress levels. Mobile apps that address the specific needs of hospitalized pregnant women are lacking and there is a great need for low-threshold therapy options. Further efforts should be made to implement a routine screening for peripartum mental disorders in maternity care, which may also enhance destigmatization. Implementing this recommendation would place an enormous burden on the German health care system, which already has a shortage of mental health professionals. Therefore, the findings of our study underscore the need for larger randomized trials to investigate the effectiveness of implementing low-cost electronic programs for pregnant women with depression and anxiety disorders.

## Data availability statement

The raw data supporting the conclusions of this article will be made available by the authors, without undue reservation.

## Ethics statement

The studies involving human participants were reviewed and approved by the Ethical Committee of the University of Heidelberg (S-744/2018). The patients/participants provided their written informed consent to participate in this study.

## Author contributions

CS collected the data, conducted the interviews, performed the analysis, and wrote the manuscript. MG and SW revised the work critically and also wrote the manuscript. SW conceived the original idea, designed and planned the study, coordinated the process, and has the scientific responsibility. MM helped with data curation and collection. JG and SZ planned the psychotherapeutic elements of the eMBI. All authors participated in the design, conception of the study, contributed to final manuscript revision, and approved the submitted version.

## Funding

The authors declare that the main study Mind: Pregnancy was funded by the Federal Joint Committee (01NVF17034). The funder was not involved in the study design, collection, analysis, interpretation of data, the writing of this article or the decision to submit it for publication.

## Conflict of interest

The authors declare that the research was conducted in the absence of any commercial or financial relationships that could be construed as a potential conflict of interest.

## Publisher's note

All claims expressed in this article are solely those of the authors and do not necessarily represent those of their affiliated organizations, or those of the publisher, the editors and the reviewers. Any product that may be evaluated in this article, or claim that may be made by its manufacturer, is not guaranteed or endorsed by the publisher.

## Abbreviations

CBT: cognitive behavioral therapy
eMBI: electronic mindfulness-based intervention
MBI: mindfulness-based intervention
PPD: postpartum depression.

## References

[1] Wallwiener S Goetz M Lanfer A Gillessen A Suling M Feisst M. Epidemiology of mental disorders during pregnancy and link to birth outcome: a large-scale retrospective observational database study including 38,000 pregnancies. Arch Gynecol Obstet. (2019) 299:755–63. 10.1007/s00404-019-05075-2 30734864

[2] Almeida M Shrestha AD Stojanac D Miller LJ. The impact of the Covid-19 pandemic on women's mental health. Arch Womens Ment Health. (2020) 23:741–8. 10.1007/s00737-020-01092-2 33263142 PMC7707813

[3] Lebel C MacKinnon A Bagshawe M Tomfohr-Madsen L Giesbrecht G. Elevated depression and anxiety symptoms among pregnant individuals during the Covid-19 pandemic. J Affect Disord. (2020) 277:5–13. 10.1016/j.jad.2020.07.126 32777604 PMC7395614

[4] Hamidia A Kheirkhah F Faramarzi M Basirat Z Ghadimi R Chehrazi M. Depressive symptoms and psychological distress from antenatal to postnatal period in women with high-risk pregnancy: a prospective study during the Covid-19 pandemic. Indian J Psychiatry. (2021) 63:536–42. 10.4103/indianjpsychiatry.indianjpsychiatry_1272_20 35136249 PMC8793707

[5] Brandon A Trivedi MH Hynan SL Miltenberger PD Labat BD Rifkin JB. Prenatal depression in women hospitalized for obstetric risk. J Clin Psychiatry. (2008) 69:635–43. 10.4088/JCP.v69n0417 18312059 PMC2680504

[6] Weidner K Bittner A Junge-Hoffmeister J Zimmermann K Siedentopf F Richter J. A Psychosomatic intervention in pregnant in-patient women with prenatal somatic risks. J Psychosom Obstet Gynaecol. (2010) 31:188–98. 10.3109/0167482X.2010.497233 20586556

[7] Tsakiridis I Bousi V Dagklis T Sardeli C Nikolopoulou V Papazisis G. Epidemiology of antenatal depression among women with high-risk pregnancies due to obstetric complications: a scoping review. Arch Gynecol Obstet. (2019) 300:849–59. 10.1007/s00404-019-05270-1 31422459

[8] Toscano M Royzer R Castillo D Li D Poleshuck E. Prevalence of depression or anxiety during antepartum hospitalizations for obstetric complications: a systematic review and meta-analysis. Obstet Gynecol. (2021) 137:881–91. 10.1097/AOG.0000000000004335 33831928 PMC8087456

[9] Fairbrother N Young AH Zhang A Janssen P Antony MM. The prevalence and incidence of perinatal anxiety disorders among women experiencing a medically complicated pregnancy. Arch Womens Ment Health. (2017) 20:311–9. 10.1007/s00737-016-0704-7 28032213

[10] Heaman M. Stressful life events, social support, and mood disturbance in hospitalized and non-hospitalized women with pregnancy-induced hypertension. Can J Nurs Res Arch. (1992) 24:23–37. 1464054

[11] Heaman M. Psychosocial impact of high-risk pregnancy: hospital and home care. Clin Obstet Gynecol. (1998) 41:626–39. 10.1097/00003081-199809000-00017 9742359

[12] Maloni JA. Antepartum bed rest for pregnancy complications: efficacy and safety for preventing preterm birth. Biol Res Nurs. (2010) 12:106–24. 10.1177/1099800410375978 20798159

[13] Melville JL Gavin A Guo Y Fan MY Katon WJ. Depressive disorders during pregnancy: prevalence and risk factors in a large urban sample. Obstet Gynecol. (2010) 116:1064–70. 10.1097/AOG.0b013e3181f60b0a 20966690 PMC3068619

[14] Lancaster CA Gold KJ Flynn HA Yoo H Marcus SM Davis MM. Risk factors for depressive symptoms during pregnancy: a systematic review. Am J Obstet Gynecol. (2010) 202:5–14. 10.1016/j.ajog.2009.09.007 20096252 PMC2919747

[15] Biaggi A Conroy S Pawlby S Pariante CM. Identifying the women at risk of antenatal anxiety and depression: a systematic review. J Affect Disord. (2016) 191:62–77. 10.1016/j.jad.2015.11.014 26650969 PMC4879174

[16] Alder J Fink N Bitzer J Hösli I Holzgreve W. Depression and anxiety during pregnancy: a risk factor for obstetric, fetal and neonatal outcome? A critical review of the literature. J Matern Fetal Neonatal Med. (2007) 20:189–209. 10.1080/14767050701209560 17437220

[17] Stein A Pearson RM Goodman SH Rapa E Rahman A McCallum M. Effects of perinatal mental disorders on the fetus and child. Lancet. (2014) 384:1800–19. 10.1016/S0140-6736(14)61277-0 25455250

[18] Grigoriadis S Graves L Peer M Mamisashvili L Tomlinson G Vigod SN. Maternal anxiety during pregnancy and the association with adverse perinatal outcomes: systematic review and meta-analysis. J Clin Psychiatry. (2018) 79:17r12011. 10.4088/JCP.17r12011 30192449

[19] Jarde A Morais M Kingston D Giallo R MacQueen GM Giglia L. Neonatal outcomes in women with untreated antenatal depression compared with women without depression: a systematic review and meta-analysis. JAMA Psychiatry. (2016) 73:826–37. 10.1001/jamapsychiatry.2016.0934 27276520

[20] Szegda K Markenson G Bertone-Johnson ER Chasan-Taber L. Depression during pregnancy: a risk factor for adverse neonatal outcomes? A critical review of the literature. J Matern Fetal Neonatal Med. (2014) 27:960–7. 10.3109/14767058.2013.845157 24044422 PMC4812822

[21] Grote NK Bridge JA Gavin AR Melville JL Iyengar S Katon WJ. Meta-analysis of depression during pregnancy and the risk of preterm birth, low birth weight, and intrauterine growth restriction. Arch Gen Psychiatry. (2010) 67:1012–24. 10.1001/archgenpsychiatry.2010.111 20921117 PMC3025772

[22] Pearson RM Evans J Kounali D Lewis G Heron J Ramchandani PG. Maternal depression during pregnancy and the postnatal period: risks and possible mechanisms for offspring depression at age 18 years. JAMA Psychiatry. (2013) 70:1312–9. 10.1001/jamapsychiatry.2013.2163 24108418 PMC3930009

[23] Andrade SE Raebel MA Brown J Lane K Livingston J Boudreau D. Use of antidepressant medications during pregnancy: a multisite study. Am J Obstet Gynecol. (2008) 198:194.e1–5. 10.1016/j.ajog.2007.07.036 17905176

[24] Ververs T Kaasenbrood H Visser G Schobben F de Jong-van den Berg L Egberts T. Prevalence and patterns of antidepressant drug use during pregnancy. Eur J Clin Pharmacol. (2006) 62:863–70. 10.1007/s00228-006-0177-0 16896784

[25] Bakker MK Kölling P van den Berg PB de Walle HE. de Jong van den Berg LT. Increase in use of selective serotonin reuptake inhibitors in pregnancy during the last decade, a population-based cohort study from the Netherlands. Br J Clin Pharmacol. (2008) 65:600–6. 10.1111/j.1365-2125.2007.03048.x 17953715 PMC2291385

[26] Huybrechts KF Palmsten K Mogun H Kowal M Avorn J Setoguchi-Iwata S. National trends in antidepressant medication treatment among publicly insured pregnant women. Gen Hosp Psychiatry. (2013) 35:265–71. 10.1016/j.genhosppsych.2012.12.010 23374897 PMC4077674

[27] Goodman JH. Women's attitudes, preferences, and perceived barriers to treatment for perinatal depression. Birth. (2009) 36:60–9. 10.1111/j.1523-536X.2008.00296.x 19278385

[28] van Ravesteyn LM Lambregtse-van den Berg MP Hoogendijk WJ Kamperman AM. Interventions to treat mental disorders during pregnancy: a systematic review and multiple treatment meta-analysis. PLoS ONE. (2017) 12:e0173397. 10.1371/journal.pone.0173397 28358808 PMC5373816

[29] Dimidjian S Goodman S. Nonpharmacologic intervention and prevention strategies for depression during pregnancy and the postpartum. Clin Obstet Gynecol. (2009) 52:498–515. 10.1097/GRF.0b013e3181b52da6 19661764 PMC5805470

[30] O'Mahen HA Flynn HA. Preferences and perceived barriers to treatment for depression during the perinatal period. J Womens Health. (2008) 17:1301–9. 10.1089/jwh.2007.0631 18816202

[31] Goodman SH Dimidjian S Williams KG. Pregnant African American women's attitudes toward perinatal depression prevention. Cultur Divers Ethnic Minor Psychol. (2013) 19:50–7. 10.1037/a0030565 23356356

[32] Marchesi C Ossola P Amerio A Daniel BD Tonna M De Panfilis C. Clinical management of perinatal anxiety disorders: a systematic review. J Affect Disord. (2016) 190:543–50. 10.1016/j.jad.2015.11.004 26571104

[33] Tsai SJ Lee YC Yang CH Sim CB. Psychiatric consultations in obstetric inpatients. J Obstet Gynaecol Res. (1996) 22:603–7. 10.1111/j.1447-0756.1996.tb01078.x 9037952

[34] Lin HL Chou HH Liu CY Hsu SC Hsiao MC Juang YY. The role of consulting psychiatrists for obstetric and gynecologic inpatients. Chang Gung Med J. (2011) 34:57–64. 21392475

[35] Buekens P van Heeringen K Boutsen M Smekens P Mattellaer P. Depressive symptoms are often unrecognized in gynaecological practice. Eur J Obstet Gynecol Reprod Biol. (1998) 81:43–5. 10.1016/S0301-2115(98)00134-1 9846712

[36] Hübner-Liebermann B Hausner H Wittmann M. Recognizing and treating peripartum depression. Dtsch Arztebl Int. (2012) 109:419–24. 10.3238/arztebl.2012.0419 22787503 PMC3394379

[37] Marcus SM Flynn HA Blow FC Barry KL. Depressive symptoms among pregnant women screened in obstetrics settings. J Womens Health. (2003) 12:373–80. 10.1089/154099903765448880 12804344

[38] Kelly R Zatzick D Anders T. The detection and treatment of psychiatric disorders and substance use among pregnant women cared for in obstetrics. Am J Psychiatry. (2001) 158:213–9. 10.1176/appi.ajp.158.2.213 11156803

[39] Gawley L Einarson A Bowen A. Stigma and attitudes towards antenatal depression and antidepressant use during pregnancy in healthcare students. Adv Health Sci Educ Theory Pract. (2011) 16:669–79. 10.1007/s10459-011-9289-0 21431361

[40] Moore D Ayers S Drey N. A thematic analysis of stigma and disclosure for perinatal depression on an online forum. JMIR Mental Health. (2016) 3:e18. 10.2196/mental.5611 27197516 PMC4909386

[41] Khoury B Lecomte T Fortin G Masse M Therien P Bouchard V. Mindfulness-based therapy: a comprehensive meta-analysis. Clin Psychol Rev. (2013) 33:763–71. 10.1016/j.cpr.2013.05.005 23796855

[42] Li J Cai Z Li X Du R Shi Z Hua Q. Mindfulness-based therapy versus cognitive behavioral therapy for people with anxiety symptoms: a systematic review and meta-analysis of random controlled trials. Ann Palliat Med. (2021) 10:7596–612. 10.21037/apm-21-1212 34353047

[43] Dhillon A Sparkes E Duarte RV. Mindfulness-based interventions during pregnancy: a systematic review and meta-analysis. Mindfulness. (2017) 8:1421–37. 10.1007/s12671-017-0726-x 29201244 PMC5693962

[44] Goodman JH Guarino A Chenausky K Klein L Prager J Petersen R. Calm pregnancy: results of a pilot study of mindfulness-based cognitive therapy for perinatal anxiety. Arch Womens Ment Health. (2014) 17:373–87. 10.1007/s00737-013-0402-7 24449191 PMC4107206

[45] Muzik M Hamilton SE Lisa Rosenblum K Waxler E Hadi Z. Mindfulness yoga during pregnancy for psychiatrically at-risk women: preliminary results from a pilot feasibility study. Complement Ther Clin Pract. (2012) 18:235–40. 10.1016/j.ctcp.2012.06.006 23059438 PMC4336321

[46] Dimidjian S Goodman SH Felder JN Gallop R Brown AP Beck A. An open trial of mindfulness-based cognitive therapy for the prevention of perinatal depressive relapse/recurrence. Arch Womens Ment Health. (2015) 18:85–94. 10.1007/s00737-014-0468-x 25298253

[47] Miklowitz DJ Semple RJ Hauser M Elkun D Weintraub MJ Dimidjian S. Mindfulness-based cognitive therapy for perinatal women with depression or bipolar spectrum disorder. Cognit Ther Res. (2015) 39:590–600. 10.1007/s10608-015-9681-9 32063660 PMC7021274

[48] Sommers-Spijkerman M Austin J Bohlmeijer E Pots W. New evidence in the booming field of online mindfulness: an updated meta-analysis of randomized controlled trials. JMIR Ment Health. (2021) 8:e28168. 10.2196/28168 34279240 PMC8329762

[49] Spijkerman MP Pots WT Bohlmeijer ET. Effectiveness of online mindfulness-based interventions in improving mental health: a review and meta-analysis of randomised controlled trials. Clin Psychol Rev. (2016) 45:102–14. 10.1016/j.cpr.2016.03.009 27111302

[50] Maloni JA Przeworski A Damato EG. Web recruitment and internet use and preferences reported by women with postpartum depression after pregnancy complications. Arch Psychiatr Nurs. (2013) 27:90–5. 10.1016/j.apnu.2012.12.001 23540519

[51] Krusche A Dymond M Murphy SE Crane C. Mindfulness for pregnancy: a randomised controlled study of online mindfulness during pregnancy. Midwifery. (2018) 65:51–7. 10.1016/j.midw.2018.07.005 30099285

[52] Avalos LA Aghaee S Kurtovich E Quesenberry Jr C Nkemere L McGinnis MK. A mobile health mindfulness intervention for women with moderate to moderately severe postpartum depressive symptoms: feasibility study. JMIR Ment Health. (2020) 7:e17405. 10.2196/17405 33180028 PMC7691085

[53] Mendelson T McAfee C Damian AJ Brar A Donohue P Sibinga E. Mindfulness intervention to reduce maternal distress in neonatal intensive care: a mixed methods pilot study. Arch Womens Ment Health. (2018). 10.1007/s00737-018-0862-x 29872924

[54] Haga SM Drozd F Brendryen H Slinning K. Mamma Mia: A feasibility study of a web-based intervention to reduce the risk of postpartum depression and enhance subjective well-being. JMIR Res Protoc. (2013) 2:e29. 10.2196/resprot.2659 23939459 PMC3742405

[55] Lever Taylor B Cavanagh K Strauss C. The effectiveness of mindfulness-based interventions in the perinatal period: a systematic review and meta-analysis. PLoS One. (2016) 11:e0155720. 10.1371/journal.pone.0155720 27182732 PMC4868288

[56] Müller M Matthies LM Goetz M Abele H Brucker SY Bauer A. Effectiveness and cost-effectiveness of an electronic mindfulness-based intervention (Embi) on maternal mental health during pregnancy: the mindmom study protocol for a randomized controlled clinical trial. Trials. (2020) 21:933. 10.1186/s13063-020-04873-3 33203471 PMC7672841

[57] Goetz M Schiele C Müller M Matthies LM Deutsch TM Spano C. Effects of a brief electronic mindfulness-based intervention on relieving prenatal depression and anxiety in hospitalized high-risk pregnant women: exploratory pilot study. J Med Internet Res. (2020) 22:e17593. 10.2196/17593 32780023 PMC7448174

[58] Elo S Kyngäs H. The Qualitative Content Analysis Process. J Adv Nurs. (2008) 62:107–15. 10.1111/j.1365-2648.2007.04569.x 18352969

[59] Hsieh HF Shannon SE. Three approaches to qualitative content analysis. Qual Health Res. (2005) 15:1277–88. 10.1177/1049732305276687 16204405

[60] Graneheim UH Lundman B. Qualitative content analysis in nursing research: concepts, procedures and measures to achieve trustworthiness. Nurse Educ Today. (2004) 24:105–12. 10.1016/j.nedt.2003.10.001 14769454

[61] Mays N Pope C. Qualitative research in health care. Assessing quality in qualitative research. BMJ. (2000) 320:50–2. 10.1136/bmj.320.7226.50 10617534 PMC1117321

[62] Byatt N Hicks-Courant K Davidson A Levesque R Mick E Allison J. Depression and anxiety among high-risk obstetric inpatients. Gen Hosp Psychiatry. (2014) 36:644–9. 10.1016/j.genhosppsych.2014.07.011 25149040 PMC4399814

[63] Hall HG Beattie J Lau R East C Anne Biro M. Mindfulness and perinatal mental health: a systematic review. Women Birth. (2016) 29:62–71. 10.1016/j.wombi.2015.08.006 26346905

[64] Schumer MC Lindsay EK Creswell JD. Brief mindfulness training for negative affectivity: a systematic review and meta-analysis. J Consult Clin Psychol. (2018) 86:569–83. 10.1037/ccp0000324 29939051 PMC6441958

[65] Baer R Crane C Miller E Kuyken W. Doing no harm in mindfulness-based programs: conceptual issues and empirical findings. Clin Psychol Rev. (2019) 71:101–14. 10.1016/j.cpr.2019.01.001 30638824 PMC6575147

[66] Statistisches Bundesamt (Destatis). Durchschnittliches Alter Der Mutter Bei Der Geburt Des Kindes 2020 (Biologische Geburtenfolge) Nach Bundesländern. (2021). Available online at: https://www.destatis.de/DE/Themen/Gesellschaft-Umwelt/Bevoelkerung/Geburten/Tabellen/geburten-mutter-alter-bundeslaender.html (accessed March 03, 2022).

[67] Bennett HA Einarson A Taddio A Koren G Einarson TR. Prevalence of depression during pregnancy: systematic review. Obstet Gynecol. (2004) 103:698–709. 10.1097/01.AOG.0000116689.75396.5f 15051562

[68] Mukherjee S Trepka MJ Pierre-Victor D Bahelah R Avent T. Racial/Ethnic disparities in antenatal depression in the United States: a systematic review. Matern Child Health J. (2016) 20:1780–97. 10.1007/s10995-016-1989-x 27016352

[69] Moser A Korstjens I. Series: practical guidance to qualitative research. part 3: sampling, data collection and analysis. Eur J Gen Pract. (2018) 24:9–18. 10.1080/13814788.2017.1375091 29199486 PMC5774281

[70] Eysenbach G. The law of attrition. J Med Internet Res. (2005) 7:e11. 10.2196/jmir.7.1.e11 15829473 PMC1550631

[71] Scherer S Alder J Gaab J Berger T Ihde K Urech C. Patient satisfaction and psychological well-being after internet-based cognitive behavioral stress management (Ib-Cbsm) for women with preterm labor: a randomized controlled trial. J Psychosom Res. (2016) 80:37–43. 10.1016/j.jpsychores.2015.10.011 26721546

